# Adherence to Updated Childcare Nutrition Regulations in Colorado, United States

**DOI:** 10.3389/fpubh.2020.00102

**Published:** 2020-04-08

**Authors:** Amy A. Eyler, Cheryl R. Valko, Katherine A. Curoe, Ramya Ramadas, Jamie F. Chriqui

**Affiliations:** ^1^Prevention Research Center in St. Louis, Brown School, Washington University in St. Louis, St. Louis, MO, United States; ^2^Division of Health Policy and Administration and Institute for Health Research and Policy, School of Public Health, University of Illinois at Chicago, Chicago, IL, United States

**Keywords:** policy, childcare, nutrition, regulations, children, obesity

## Abstract

**Background:** Preschool years are an important time for shaping healthy eating behaviors. Childcare centers can be a venue for policy change for broad and sustained positive impact on healthy eating environment. The objectives of this study were to assess how self-reported current practices align with updated statewide childcare center licensing regulations in Colorado, US, and to explore correlates of adherence.

**Methods:** Using a post-test only study design, a survey was sent to all full-day, licensed childcare centers in Colorado (*N* = 1,398) with a valid street or email address. The survey included questions on allowable food and beverages, mealtime practices, and perceptions of the updated regulations. Frequencies were calculated and logistic regression models computed for a composite score of each of these factors.

**Results:** Respondents (*N* = 344) were mostly center directors, with over 8 years of experience, from urban areas. Compliance was high for most food and beverage criteria (over 90%) and all meal practices. One third participated in the federal Child and Adult Care Food Program (CACFP), and were more likely to comply with the state meal regulations than non-CACFP centers.

**Conclusion:** Although our results show high self-reported compliance, a more thorough study of the policy process would provide comprehensive evidence on effective development, enactment, and implementation of these regulations.

## Introduction

Preventing obesity in young children holds promise for reversing the epidemic of childhood obesity (1) Among 2–5 year olds in the United States, the rate of obesity has more than doubled, up from 5% in 1980, to 13.9% in 2015 (2). The increased rate in this group, as well as in older children and adolescents, has made childhood obesity one of the most serious public health challenges of the twenty first century (1, 3).

The preschool years are an important time for promoting healthy eating, as behaviors developed in this period may persist into adulthood (4, 5). An especially relevant setting for nutrition interventions in this age group is the childcare center (6). It is estimated that 80% of preschool age children in the United States spend an average of 40 h a week in out-of-home childcare (7). Because of the number of meals and snacks that children consume during their time in childcare, policies that outline healthy eating and mealtime practice guidelines can be effective and sustainable strategies to increase healthy eating (1, 6). Policies prohibiting serving sugar-sweetened beverages to children, for instance, may help them develop preferences for water or milk during mealtimes (8).

Childcare centers are governed by federal, state, and organizational policies. For example, centers may opt to participate in the Child and Adult Care Food Program (CACFP). CACFP is a federal program that reimburses centers for the cost of eligible meals and snacks served to enrolled children, targeting those children most in need (9). In response to the childhood obesity epidemic and other national child health efforts, recently updated CACFP meal pattern standards require childcare centers to serve more whole grains, a wider variety of fruits and vegetables, and less solid fats and added sugars (9). In addition to national policies, states have the opportunity to set healthy food requirements, as childcare licensing occurs at the state level. Childcare licensing requirements can be outlined in state statutes and/or regulations and the criteria for licensure varies by state.

The state of Colorado recently adopted new criteria for childcare center licensure, including changes in healthy eating requirements. Efforts began in 2015 when the Colorado Department of Human Services Office of Early Childhood and the Colorado Board of Health worked together to develop and approve the new standards. These regulations ensure that all food provided in childcare centers meets CACFP requirements (9, 10), and prohibits sugar-sweetened beverages from being served. Regulations also outline new standards for menu planning and mealtime practices. In addition to nutrition changes, these regulations also specify required amounts of physical activity and limit screen time. Past criteria for licensure did not provide explicit guidance for these nutrition and physical activity requirements. These new standards, in addition to the full list of childcare licensing regulations are the minimum health, safety, and program requirements licensed childcare providers must follow to operate legally. Several agencies offered assistance for centers needing information or other resources in order to comply with the new regulations (10). More than 100,000 children in the state will be impacted by these standards, which took effect February 2016, with full implementation required by September 2016 (11).

The American Heart Association's Voices for Healthy Kids is an advocacy program to improve or create equitable policies that will make the places children live, learn, and play healthier. As part of an evaluation of their policy advocacy efforts, the goals of the current study were to assess self-reported adherence to the updated Colorado childcare regulations related to nutrition, explore perceptions of preparedness for implementation, and identify the use of technical assistance. This study also aimed to identify correlates of reported adherence.

## Materials and Methods

### Survey Development and Administration

Changes in the state childcare licensing regulations related to nutrition informed the survey questions (see Table 1). Questions included topics such as meal preparation and length of meal times. Other mealtime practices such as use of TV/media and staff member supervision were also included. Because of the requirement changes in allowable beverages, we added questions on the practice of serving water, milk, juice, and soda. In addition to the assessment of the regulatory changes, the survey included characteristics of the respondent and the center for use as independent variables in analyses. These included title/role of respondent, ages served, ownership, staff count, and capacity. We also assessed participation in CACFP and/or the Head Start/Early Head Start Program. Similar to CACFP, Head Start (for children ages 3–4) and Early Head Start (for infants and toddlers) are federally funded program to support quality childcare for low-income families. As a proxy for socioeconomic status of overall clientele, we asked for information on average weekly rate of childcare. These characteristics were important to assess as we hypothesized that centers in urban locations, having larger center capacity size and higher income clientele would correlate with higher adherence to the regulations. The last section of the survey assessed familiarity with the regulation changes, preparation for and implementation of new regulations, additional resources needed, and use of technical assistance. Participants could choose to receive a $20 Amazon gift card for completing the survey to their preferred delivery method, either electronically or via mail. The survey was pilot tested with content experts and aligned with other childcare center food and beverage research (12, 13). One center director, three researchers, and a biostatistician previewed the survey for content and flow. The survey is available in Data Sheet 1: Survey of Child Care Sites in Colorado.

**Table 1:** Regulation language and related compliance factor assessed in the survey.

**Updated regulation**	**Compliance factor assessed**
Children who are at the center for more than 4 h, day or evening, must be offered a meal	Time at center to get a meal
Centers must not provide sugar sweetened beverages to children. These are liquids that have been sweetened with various forms of sugars that add calories and include, but are not limited to: soda, fruitades, fruit drinks, flavored milks, and sports and energy drinks	No soda or sweetened beverages, flavored milk Water availability^*^ Water testing for contaminants^*^
If 100% fruit juice, which is not a sugar sweetened beverage, is offered as part of meals and/or snacks, it must be limited to no more than twice per week	Juice served twice or less per week
The size of servings must be suitable for the child's age and appetite, and sufficient time must be allowed so that meals are unhurried	Time for meals
Staff members must sit with the children and encourage them to try a variety of food served. During meals, children should be encouraged to engage in conversation and to express their independence	Staff supervision Staff eats lunch with children^*^ Encourage new food Encourage conversation
Meal menus must be planned at least 1 week in advance, dated, and posted in a place visible to parents. After use, menus must be filed and retained for 3 months. Records must be available for periodic review and evaluation	Advance menu planning Filing of menu
All television, recorded media, computer, tablet and media devices are prohibited during snack or meal times	No screen time during meals

* *Not in regulations but assessed in survey*.

### Sample

The sample for the survey was all licensed, full-day childcare centers in the state of Colorado that enroll infants and children ages 0–5. We used a 2015 list (*N* = 1,441) of center names and addresses available from the state's early childhood website (https://data.colorado.gov/Early-childhood/Colorado-Licensed-Child-Care-Facilities-Report/a9rr-k8mu/data). Center contact information was obtained through publicly available data on childcare center webpages. Fourteen centers were no longer open for business at the time of data collection. We found valid email addresses for 963 centers and used street addresses to contact the remaining 435 centers via US Postal Service. Twenty-nine centers had neither valid emails nor street addresses, resulting in a total *N* = 1,398 for email and postal surveys. An invitation to participate was sent along with a project information page describing the study. The information page stated the survey was voluntary, questions could be skipped, and that the data would only be used in aggregate. Participants were asked to provide an email address if they wished to receive the incentive. The Institutional Review Board at Washington University in St. Louis approved this study.

### Data Collection

Data were collected February-April 2018, which was 16 months after the required date of September 2016 for compliance with updated regulations. Email participants received one initial email inviting them to participate in the study and one reminder each week for 4 weeks. Only non-responders received reminder emails. Each email contained a link to the online survey (using the online survey platform Qualtrics.com) and consent information. Centers lacking email addresses received an introductory letter, consent information, the survey, and a self-addressed, postage-paid return envelope. No reminders were sent to those receiving postal surveys.

### Analysis

Data from the online survey platform was imported into a Stata Version 15 database (Statacorp LLC, College Station, Texas). The research team manually entered data from the mailed surveys into this database. Frequencies of responses, means, and standard deviations were calculated. Two multiple logistic regression models were computed as part of our analyses. For each model, correlates were center capacity, rural urban commuting area (RUCA) classification (14), CACFP status (yes/no), status as independent center vs. corporate owned/franchise, and average weekly rate for children 2–5. A binomial logistic regression was computed individually against each correlate. Significant correlates (*p* < 0.05) were put into a larger, adjusted model. Regression diagnostics (Link-tests, correlations, variance inflation projections, and tolerance) were run on the final adjusted models. In the first model, the outcome variable was compliance with the regulations. Since there was little variability in the data set, as most centers reported adherence to the new regulations, aggregate adherence measures were created for meals and drinks, and separated by the age of children served (<2 years and 2+ years). Since none of the centers met all four drink variables, meeting three out of the four regulations was compared to meeting two or less regulations. The second regression model was based on implementation. The outcome variable for this model was readiness to implement updates regulations (i.e., very prepared to implement vs. somewhat/not at all prepared).

## Results

We received 256 completed online surveys and 88 completed paper surveys for 344 responses (response rate of 25%). This response rate is comparable to other recent early childcare surveys (12, 13, 15, 16). There was no significant difference in the size of center, CACFP participation, or geographic location between responding and non-responding centers. Table 2 depicts general characteristics of respondents and centers. Most respondents were Center Directors or Assistant Directors (64.1%) with a mean of 8.7 years (SD = 8.5) in their current role. About one-third of centers participated in CACFP (32.7%). Most centers were in urban locations (74.6%) and had an average of 17 staff on site (SD = 17.8). The average weekly rate for children <2 years of age was $309, and $319 for children aged 2–5. The average time (in hours) a child must be at the center to receive a meal was 2.5 (SD = 2.1). Mealtimes were an average of 36 min for children aged 2–5, and 34 min for school age children.

**Table 2:** General characteristics of Colorado Childcare Regulations Survey participants.

**Variable**	***N* (%)**
Role at current child care site (*n* = 423, actual *n* = 341)^*^	
Corporate/Sponsor	11 (2.6)
Center Owner/Franchisee	56 (13.2)
Center Director/Assistant Director	263 (62.2)
Teacher	52 (12.3)
Other	41 (9.7)
Participates in CACFP (*n =* 340)	
Yes	111 (32.7)
No	218 (64.1)
Don't know/not sure	11 (3.2)
Participates in Early Head Start Program (*n =* 343)	
Yes	20 (5.83)
No	322 (93.9)
Don't know/not sure	1 (0.29)
Participates in Head Start Program (*n =* 342)	
Yes	25 (7.3)
No	317 (92.7)
Don't know/not sure	0 (0.0)
Corporate-owned (*n =* 342)	
Yes	40 (11.7)
No	299 (87.4)
Don't know/not sure	3 (0.9)
Franchise-owned (*n =* 342)	
Yes	7 (2.1)
No	334 (97.7)
Don't know/not sure	1 (0.3)
RUCA classification (*n =* 236)	
Urban	176 (74.6)
Large Rural City	21 (8.90)
Small Rural Town	22 (9.32)
Isolated Small Rural Town	17 (7.20)
Age groups served (*n =* 648, actual *n =* 342)**	
<2	185 (28.6)
Ages 2–5	330 (50.9)
School age children	133 (20.5)
Meal preparation for site (*n =* 337)	
Corporate Office	14 (3.5)
Food program/CACFP/CCFP Sponsor	28 (7.1)
Center Director	92 (23.2)
On-site Kitchen/Food Manager/Cook	128 (32.3)
Meal Planning Service	6 (1.5)
Other	123 (31.1)
Don't know/not sure	5 (1.3)
Total capacity for school aged children	207.1 (1534.7)
Weekly rate for children <2 years ($)	309.0 (237.2)
Weekly rate for children aged 2–5 years ($)	319.4 (1016.3)

* *RUCA, Rural Urban Commuting Area classification*.

*n may vary due to allowing for multiple responses to questions. For example, a respondent may have multiple roles at the center, or they may serve more than one age group category.

*The term “actual” means number of unique respondents*.

*CACFP and Head Start/Early Head Start are federal programs partnering with childcare centers to provide assistance to low-income families of infants, toddlers, and young children*.

Overall self-reported adherence with the updated regulations was high for respondents of our survey (see Table 3). Most centers reported meeting the requirement of at least 1 week advance meal planning (73.1%) and keeping menus on file for at least 3 months (86.3%). Ninety-five percent of centers had staff sit with children when eating. Although not a requirement, we asked if staff members eat lunch with the children, and less than half reported doing so (41.1%). All respondents encouraged children to engage in conversation during meals, 98.5% indicated that staff encouraged children to try a variety of food during meals, and 97.6% reported prohibiting television or videos during mealtime.

**Table 3:** Number and percentage of self-reported food environment regulation adherence.

	***N* (%)**
Advance meal planning (*n =* 305)	
<1 week	29 (9.5)
1 week	37 (12.1)
2–3 weeks	47 (15.4)
4+ weeks	139 (45.6)
Don't know/not sure	53 (17.4)
Menus on file (*n =* 323)	
Yes	234 (72.5)
No	75 (23.2)
Don't know/not sure	14 (4.3)
Length of time menus kept on file (*n =* 233)	
<1 month	7 (3.0)
1–2 months	3 (1.3)
3 months	9 (3.9)
4+ months	192 (82.4)
Don't know/not sure	22 (9.4)
Staff members sit with children when eating (*n =* 339)	
Yes	322 (95.0)
No	16 (4.7)
Don't know/not sure	1 (0.3)
Staff members eat lunch with children (*n =* 336)
Yes	138 (41.1)
No	194 (57.7)
Don't know/not sure	4 (1.2)
TV/video allowed during meal time (*n =* 339)	
Yes	8 (2.4)
No	331 (97.6)
Don't know/not sure	0 (0.0)
Staff encourage children to try variety of food (*n =* 336)	
Yes	331(98.5)
No	2(0.6)
Don't know/not sure	3(0.9)
Children encouraged to engage in conversation (*n =* 339)	
Yes	338 (99.7)
No	0
Don't know/not sure	1 (0.3)
	**Mean (SD)**
Time (in hours) a child must be at center to receive meal	2.5 (2.1)
Meal times (in minutes) for children aged 2–5	35.7 (14.3)
Meal times (in minutes) for school age children	34.2 (11.0)

*variance in n due to skip patterns in survey questions.

There were several updated regulations relating to allowable drinks served at childcare centers (see Table 4). Almost all of the centers had clean, fresh water available (99.4%), and 71.1% reported regularly testing water for contaminants. No center indicated allowing soda, and most never served fruit drinks (96.6%). The percentage of centers serving flavored milk once or more a day was 11.5% for children ages 2–5 and 13.7% for school aged children.

**Table 4:** Self-reported adherence to regulations related to beverages served in Colorado childcare centers.

	**Children aged 2–5 yrs**	School aged^*^
Type of milk served	(*N* = 322)	(*n =* 128)
Whole milk	58 (16.8)	8 (5.7)
Reduced-Fat (2%) milk	104 (30.1)	47 (33.3)
Low Fat (1%) milk	126 (36.5)	63 (44.7)
Fat-free milk	25 (7.3)	11 (7.8)
Don't know/not sure	32 (9.3)	12 (8.5)
Frequency of serving flavored milk	(*n =* 322)	(*n =* 131)
Never	285 (88.5)	113 (86.3)
Once a day	15 (4.7)	11 (8.4)
Twice a day	12 (3.7)	2 (1.5)
More than twice a day	4 (1.2)	2 (1.5)
Don't know/not sure	6 (1.9)	3 (2.3)
Frequency of serving 100% juice	(*n =* 324)	(*n =* 131)
Never	240 (74.1)	92 (70.2)
1 time a week	46 (14.2)	24 (18.3)
2 times a week	27 (8.3)	11 (8.4)
3+ times a week	7 (2.2)	3 (2.3)
Don't know/not sure	4 (1.2)	1 (0.8)
Frequency of serving fruit drinks	(*n =* 323)	(*n =* 132)
Never	312 (96.6)	128 (97.0)
1 time a week	6 (1.9)	1(0.8)
2 times a week	0	0
3+ times a week	3 (0.9)	2 (1.5)
Don't know/not sure	2 (0.6)	1 (0.8)
Frequency of serving any regular soda	(*n =* 325)	(*n =* 132)
Never	325 (100.0)	131 (99.2)
1 time a week	0	1 (0.8)
2 times a week	0	0
3+ times a week	0	0
Don't know/not sure	0	0
Clean, fresh water available	(*n =* 343)	
Yes	341 (99.4)	
No	2 (0.6)	
Don't know/not sure	0	
Water tested for contaminants	(*n =* 322)	
Yes	244 (71.1)	
No	37 (10.8)	
Don't know/not sure	62 (18.1)	

* *School aged is a term for students who enroll in school. They may be enrolled in childcare simultaneously for before- or afterschool care*.

*Variance in number of respondents due to skip patterns in survey questions*.

Few centers (12.8%) reported not being familiar with the Colorado childcare center regulation updates, and most indicated being somewhat or very much prepared for implementation (96.4%) (see Table 5). About a third (31.5%) indicated that they began implementation of the updated regulations prior to February 2016, and 41.3% reported that no changes were needed because their practices already met the updates. Only 9.2% of respondents indicated that their centers received technical assistance in order to meet the requirements of the updated regulations.

**Table 5:** Perceptions of regulation preparedness.

	***N* (%)**
Familiar with Colorado child care regulations (*n =* 344)	
Yes	284 (82.6)
No	44 (12.8)
Don't know/not sure	16 (4.7)
Site prepared to make changes based on regulations (*n =* 284)	
Very much	202 (71.1)
Somewhat	69 (24.3)
Not at all	4 (1.4)
Don't know/not sure	9 (3.2)
Time when state regulations implemented (*n =* 283)	
Prior to February 2016	89 (31.5)
March 2016-December 2016	44 (15.6)
January 2017 or later	17 (6.0)
No changes were needed	117 (41.3)
Don't know/not sure	16 (5.7)
Changes in regulations required more work and resources—staffing (*n =* 283)	
Very much	13 (4.6)
Somewhat	70 (24.7)
Not at all	178 (62.9)
Don't know/not sure	22 (7.8)
Changes in regulations required more work and resources—kitchen facilities (*n =* 272)	
Very much	14 (5.2)
Somewhat	71 (26.1)
Not at all	163 (59.9)
Don't know/not sure	24 (8.8)
Needed to make changes related to nutrition due to regulations (*n =* 282)	
Very much	10 (3.6)
Somewhat	54 (19.2)
Not at all	205 (72.7)
Don't know/not sure	13 (4.6)
Technical assistance received to implement changes (*n =* 283)	
Yes	26 (9.2)
No	243 (85.9)
Don't know/not sure	14 (5.0)

There were few significant results from the regression models (see Table 6). The only variables emerging significant in binomial logistic regression were CACFP status and RUCA, which were used in the adjusted regression models. Childcare centers in isolated rural towns had 2.57 higher odds of complying with all meal regulations compared to those in urban areas, but this finding did not remain significant in the adjusted model (AOR 2.23; 95% CI 0.82–6.08). Centers participating in CACFP were almost four times as likely to meet all seven meal regulations than centers not participating in this federal program, and these odds remained statistically significant in the adjusted regression model (AOR 3.87; 95% CI 2.38–6.31). Independent status, total capacity, or weekly rate did not emerge as significant correlates. For compliance with three drink regulations as the outcome vs. meeting less than three drink regulations, odds ratios for total capacity and weekly rate for 2–5 year olds were statistically significant in the adjusted model. For every one unit (one dollar) increase in the weekly rate for 2–5 year olds, the odds of complying will three regulations increased by 1.006 (95% CI: 1.002–1.009). None of the odds ratios for the outcome variables related to readiness to implement were statistically significant; therefore, an aggregate model was not computed.

**Table 6:** Regression models for updated regulation adherence.

	**Six or less regulations met**	**All 7 regulations met**	**Unadjusted OR (95% CI)**	**Adjusted OR (95% CI)**
	***n*** **(%)**			
**Model 1. Characteristics associated with adherence with meal regulations**				
CACFP status			3.95 (2.44, 6.40)^**^	3.87 (2.38, 6.31)^**^
Yes	42 (21.4)	69 (51.9)		
No	154 (78.6)	64 (48.1)		
RUCA Status				
Urban	163 (78.7)	103 (76.9)		
Large Rural City	15 (7.3)	9 (6.7)	0.95 (0.40, 2.25)	0.92 (0.37, 2.31)
Small rural town	21 (10.1)	9 (6.7)	0.68 (0.30, 1.54)	0.66 (0.28, 1.56)
Isolated rural town	8 (3.9)	13 (9.7)	2.57 (1.03, 6.42)^*^	2.23 (0.82, 6.08)
Independent			0.78 (0.47, 1.30)	
Yes	161 (78.2)	98 (73.7)		
No	45 (21.8)	35 (23.6)		
	**Mean (SD)**			
Total Capacity	88.3 (94.82)	98.3 (69.72)	1.00 (1.00, 1.00)	
Weekly rate for age 2–5 years	232.7 (215.69)	249.6 (207.79)	1.00 (1.00, 1.00)	
	**Less than 3 regulations met**	**3 regulations met**	**Unadjusted OR (95% CI)**	**Adjusted OR (95% CI)**
	***n*** **(%)**			
**Model 2: Characteristics associated with compliance with Drink regulations**				
CACFP status			1.05 (0.58, 1.90)	
Yes	20 (32.3)	87 (33.1)		
No	42 (67.7)	174 (66.7)		
RUCA Status				
Urban	53 (80.3)	207 (77.2)		
Large Rural City	4 (6.1)	20 (7.5)	1.28 (0.42, 3.90)	
Small rural town	4 (6.1)	25 (9.3)	1.60 (0.53, 4.80)	
Isolated rural town	5 (7.6)	16 (6.0)	0.82 (0.29, 2.34)	
Independent			1304 (0.55, 1.95)	
Yes	49 (75.4)	204 (76.1)		
No	16 (24.6)	64 (23.9)		
	**Mean (SD)**			
Total Capacity	122.59 (137.17)	86.00 (66.11)	1.00 (0.99, 1.00)^*^	0.995 (0.992, 0.999)^*^
Weekly rate for age 2–5 years	164.20 (101.38)	253.38 (223.96)	1.00 (1.00, 1.01)^*^	1.006 (1.002, 1.009)^*^

* *p < 0.05*;

** *p < 0.001*.

## Discussion

Policies are an important element in influencing early eating behavior and childhood obesity prevention (1, 3, 17). Policies and regulations for child care are especially relevant due to their broad reach and sustainability (1, 6). However, enactment of the policies must be followed by correct interpretation and timely implementation to achieve the intended outcome (18). There was very high self-reported adherence with all components of the new Colorado childcare regulations, and this may be due to several factors. First, Colorado is one of the healthiest states in the nation. It has the lowest prevalence of adult obesity (19) and ranks second lowest in childhood obesity rates for 2–4 year olds enrolled in the Women, Infants, and Children (WIC) federal nutrition assistance program (20). These rates may be due, in part, to policy priorities for health and thus sets the tone for a culture of adherence within the state. In addition, national publicity and programming to reduce childhood obesity may have helped increase awareness of the changes needed. For example, updated federal requirements for food and snacks served in public schools (20), the presidential priority on childhood obesity prevention (21), and the increasing evidence of detrimental effects of sugar-sweetened beverages may have primed the childcare centers for the regulatory changes in Colorado (20, 22). Many federal requirements for centers receiving CACFP funds align with the Colorado regulations (e.g., prohibiting sugar-sweetened beverages) (8). Centers participating in CACFP would likely already be compliant as indicated by our findings that they were four times more likely to meet all meal regulations than non-CACFP sites. As such, over 40% of participants reported needing no changes because existing policies already met the new requirements. However, this still leaves room for improvement in non-CACFP centers. Second, we assessed aspects of the regulations almost 16 months after required implementation. This gave the centers sufficient time to learn about licensure changes and make use of technical assistance provided by the state and advocacy organizations. Preparation by the centers to adhere to the new regulation may have decreased the need for technical assistance related to implementation. Over 95% of the participants in this study reported being prepared to implement the new regulations, and only 9.2% reported receiving technical assistance. Perhaps a more explicit definition of technical assistance and assessing a timeframe in which it was sought or used would have shown different results. Future studies should include implementation assessment throughout the course of the policy process; from development, enactment, and beyond. This would provide insight on when technical assistance would be most beneficial.

Although our findings showed high self-reported adherence, there is room for improvement. None of respondents allowed regular soda to be served to children, but over 10% indicated serving flavored milk, which contains a significant amount of added sugar. Developing materials for enhanced awareness of specific allowable foods may be helpful to reach 100% compliance.

Positive mealtime experiences in youth can help shape healthy eating behaviors later in life (4, 5, 23). The updated Colorado regulations included several aspects of positive mealtimes including adequate time for eating, no television/video, encouraging conversation, and promoting new foods. Over 95% of participants reported mealtime practices outlined in the regulations were being implemented. Additionally, role modeling behavior is important to developing healthy eating behaviors (16, 24). Even though requiring staff to eat meals with the children was not part of the licensing regulations, we inquired about this practice in the survey. Fewer than half of respondents reported doing so. More research is needed on the impact of childcare staff modeling healthy eating on the development of eating habits in young children.

There is evidence that characteristics of childcare centers such as location and CACFP participation may influence implementation of nutrition policies (25–28). We theorized that larger, urban childcare centers serving higher income populations would have higher adherence to the regulations. However, our logistic regression models resulted in few statistically significant findings. This points to equitable practices in centers across the state and adherence even without CACFP participation.

Policies governing childcare nutrition practices have an important role in child obesity prevention (1, 29). Monitoring for adoption and compliance of these policies is needed to facilitate intended policy impact. While self-reported compliance was high in our Colorado study, results from other US studies vary. For example, a cross-sectional study of Rhode Island childcare center directors also showed comparable compliance with prohibition of sugar-sweetened beverages (30). However, results from a study of adherence in Nebraska childcare centers found sub-optimal reported implementation (80% or less) of several nutrition best practices (25). A Delaware assessment showed compliance ranging from 35.6% for water availability outside to 88% for juice type (31). National studies on federal CACFP regulations also yield mixed self-reported compliance (12, 13). Continuing to strengthen state and national standards, and comprehensively monitoring childcare centers compliance with those standards will enhance the potential for improving childhood obesity prevalence.

Although findings from this study show healthy food environments and practices in Colorado childcare centers, several limitations should be noted. First, Colorado is one of the healthiest states in the United States, particularly related to obesity. Healthy nutrition practices may have been standard prior to the regulation changes. Our post-test only study design did not allow for assessment of change in practices before and after the new regulation. Having baseline data would have strengthened evidence of policy implementation, but we were constrained by timing of the project. We developed the survey to assess variables unique to the Colorado regulations, and thus could not rely on existing validated measures. Best practices for survey research (32, 33) including the use of incentives, follow-up, and the use of email and mail surveys resulted in a response rate of 25%, and the participants may not be representative of all childcare centers in the state, thus generalization is limited. However, when we compared respondent sites to non-respondent sites from the sample list, there were no significant differences in the center characteristics of size of center, CACFP participation, or geographic location. Additionally, social desirability bias may have influenced responses because we were assessing implementation of requirements for licensure. Another limitation was although the survey was sent to the director, it may have been delegated to other staff. We did not collect information on perception of importance by the director, and delegation may have been influenced by low acceptability. These regulations apply to only licensed early childcare centers and do not include licensed home-based day care. There were 1,509 licensed home-based centers in Colorado in 2014, with the capacity to serve almost 9,000 children. Because of the prevalence of home-based day care, this is an important area for future study. In spite of these limitations, our study contributes to knowledge on the potential for policy to influence healthy food practices and environment in childcare centers.

## Conclusion

Policies and regulations for childcare centers have the potential to increase exposure to healthy eating environments in early childhood. Although our results show high self-reported preparedness, adherence to the updated regulations and low use of technical assistance, a more thorough study of the policy process would provide comprehensive evidence on effective development, enactment, and implementation of these regulations.

## Data Availability Statement

The datasets generated for this study are available on request to the corresponding author.

## Ethics Statement

This study was exempt per 45 CFR 46.101(b) (2) of the Federal Policy for the Protection of Human Subjects (Common Rule). The protocol was approved by the Washington University in St. Louis Institutional Review Board.

## Author Contributions

Conceptualization: AE and JC. Methodology: AE, CV, and JC. Analysis: RR and KC. Writing-original draft preparation: AE, KC, and RR. Writing-review and editing: AE, CV, RR, KC, and JC. Project administration: CV. Significantly contributed to the development of this manuscript: All authors.

### Conflict of Interest

The authors declare that the research was conducted in the absence of any commercial or financial relationships that could be construed as a potential conflict of interest.
