# Supplementary material for: Adherence to Updated Childcare Nutrition Regulations in Colorado, United States
Source: Front Public Health. 2020 Apr 8;8:102. doi: 10.3389/fpubh.2020.00102 (PMC7156549; doi:10.3389/fpubh.2020.00102)
Supplement: Supplementary file 1 [file Data_Sheet_1.pdf]

PREVENTION RESEARCH CENTER in St. Louis
Promoting Healthy Communities

Survey of Child Care Sites in Colorado

Section A. Introductory questions.

1. What is the name of your child care site? If you are a sponsor, please indicate the site for which you are completing the survey. (optional)

____

2. What email address should we use to send your $20 Amazon gift card to for completing the survey? (optional)

Email: _________________________________

☐ I would like to opt out of the gift card

3. What is your role at your current child care site?

(Select all that apply.)

☐ Corporate/ Sponsor
☐ Center Owner/ Franchisee
☐ Center Director/ Assistant Director
☐ Teacher
☐ Other (please indicate) _____________________

4. How long have you been in your role at your current child care site?

_____________________ years

Section B. About your child care site.

5. Does your child care site participate in the U.S. Department of Agriculture's Child and Adult Care Food Program (CACFP) also known as the Federal Food Program or Child Care Food Program (CCFP)?

☐ Yes
☐ No
☐ I don't know

6. Does your site participate in the <u>Early</u> Head Start Program?

☐ Yes
☐ No
☐ I don't know

7. Does your site participate in the Head Start Program?

☐ Yes
☐ No
☐ I don't know

8. Is your site corporate owned?

☐ Yes
☐ No
☐ I don't know

9. Is your site owned by a franchisee?

☐ Yes
☐ No
☐ I don't know

10. How many staff are employed at your site? Staff includes anyone on your payroll (including yourself).

____

11. What age groups does your site serve? (Select all that apply.)

☐ Children under 2
☐ Children ages 2-5
☐ School age children
☐ I don't know

12. What is the total enrollment capacity for your site?

______________________ total children

13. What is your site's total enrollment capacity for each age category?

Children under 2 years: _______________________

Children ages 2-5 years: ______________________

School age children: _________________________

14. What is the average weekly rate per child for full time children under 2 years old to attend your child care site without scholarship or financial aid?

$__________________ for full time children under 2 years

☐ N/A

15. What is the average weekly rate per child for full time children ages 2-5 to attend your child care site without scholarship or financial aid?

$__________________ for full time children 2-5 years old

☐ N/A

Section C. About meals at your site.

16. How many hours must a child be at your center to be offered a meal?

________________hours

17. How long are meal times for 2-5 years olds?

________________minutes

☐ N/A

18. How long are meal times for school age children?

________________minutes

☐ N/A

19. Who prepares the menus for your site? (Select all that apply.)

☐ Corporate Office
☐ Food Program/CACFP/CCFP Sponsor
☐ Center Director
☐ On-site Kitchen/Food Manager/Cook
☐ Meal Planning Service (e.g. Minute Menu, Procare Software)
☐ Other (please indicate) ________________________
☐ I don't know

20. How far in advance are menus prepared?

☐ <1 week
☐ 1 week
☐ 2-3 weeks
☐ 4+ weeks
☐ I don't know

21. Do you keep menus on file?

☐ Yes
☐ No → If NO, go to Question #23
☐ I don't know → If I DON'T KNOW, go to Question #23

22. For how long do you keep menus on file?

☐ <1 month
☐ 1 month
☐ 2-3 months
☐ 4+ months
☐ I don't know

Section C.1. Mealtime practices.

**23. Staff members must sit with the children while they eat.**	Yes	No	I don't know
**24. Television and/or video viewing is permitted during meal times.**	Yes	No	I don't know
**25. Staff members eat their own lunch with the children.**	Yes	No	I don't know
**26. Staff members encourage the children to try a variety of food served.**	Yes	No	I don't know
**27. During meals, children are encouraged to engage in conversations.**	Yes	No	I don't know

Section D.1. Screen time and physical activity for children under 2.

☐ My site does not serve children under 2. → If checked, go to Question #31.

28. In an average week, how much time do children under 2 spend watching television and/or videos?

________________hours

29. On an average day, how much time do children under 2 spend using a computer and/or tablet?

☐ None
☐ Less than 30 minutes
☐ 30 minutes
☐ More than 30 minutes
☐ I don't know

30. How often is outdoor play provided for children under 2? (weather permitting)

☐ Never
☐ 1 day per week
☐ 2 days per week
☐ 3 days per week
☐ 4 days per week
☐ Daily
☐ I don't know

Section D.2. Screen time and physical activity for children ages 2-5.

☐ My site does not serve children ages 2-5. ⟶ If checked, go to Question #35.

31. In an <u>average week</u>, how much time do <u>children ages 2-5</u> spend watching television and/or videos?

________________hours

32. On an <u>average day</u>, how much time do <u>children ages 2-5</u> spend using a computer and/or tablet?

☐ None
☐ Less than 30 minutes
☐ 30 minutes
☐ More than 30 minutes
☐ I don't know

33. On an <u>average day</u>, how much time do <u>children ages 2-5</u> spend in active play or physical activity? (These could be structured activities such as staff leading a dance activity or unstructured play where children are moving around.)

☐ None
☐ Less than 30 minutes
☐ 30-59 minutes
☐ 60 or more minutes
☐ I don't know

34. How often is outdoor play provided for <u>children ages 2-5</u>? (weather permitting)

☐ Never
☐ 1 day per week
☐ 2 days per week
☐ 3 days per week
☐ 4 days per week
☐ Daily
☐ I don't know

Section D.3. Screen time and physical activity for school age children.

☐ My site does not serve school age children. ⟶ If checked, go to Question #39.

35. In an <u>average week</u>, how much time do <u>school age children</u> spend watching television and/or videos?

________________hours

36. On an <u>average day</u>, how much time do <u>school age children</u> spend using a computer and/or tablet?

☐ None
☐ Less than 30 minutes
☐ 30 minutes
☐ More than 30 minutes
☐ I don't know

37. On an <u>average day</u>, how much time do <u>school age children</u> spend in active play or physical activity? (These could be structured activities such as staff leading a dance activity or unstructured play where children are moving around.)

☐ None
☐ Less than 30 minutes
☐ 30-59 minutes
☐ 60 or more minutes
☐ I don't know

38. How often is outdoor play provided for <u>school age children</u>? (weather permitting)

☐ Never
☐ 1 day per week
☐ 2 days per week
☐ 3 days per week
☐ 4 days per week
☐ Daily
☐ I don't know

Section E. Food and beverages.

39. Is clean, fresh water available to children upon request throughout the day, such as a water fountain or in a cup?

☐ Yes
☐ No
☐ I don't know

40. Is the site's tap water tested for contaminants?

☐ Yes
☐ No
☐ I don't know

Section E.1. Food and beverages for children ages 2-5.

☐ My site does not serve children ages 2-5. ⟶ If checked, go to Question #46.

41. What type of milk does your site serve to children ages 2-5? (Select all that apply.)

- ☐ Whole Milk
- ☐ Reduced-Fat (2%) Milk
- ☐ Low-Fat (1%) Milk
- ☐ Fat-Free (Skim) Milk
- ☐ I don't know

42. How often does your site serve flavored milk to children ages 2-5?

- ☐ Never
- ☐ Once a day
- ☐ Twice a day
- ☐ More than twice a day
- ☐ I don't know

43. How often does your site serve 100% juice as labeled on the package to children ages 2-5 as part of a meal and/ or snack?

- ☐ Never
- ☐ 1 time per week
- ☐ 2 times per week
- ☐ 3 or more times per week
- ☐ I don't know

44. How often does your site serve fruit drinks or fruit-flavored beverages other than 100% fruit juice such as Sunny Delight, Hi-C, Kool Aid, or lemonade to children ages 2-5?

- ☐ Never
- ☐ 1 time per week
- ☐ 2 times per week
- ☐ 3 or more times per week
- ☐ I don't know

45. How often does your site serve regular soda/pop (such as Sprite, orange soda, grape soda, cola) to children ages 2-5?

- ☐ Never
- ☐ 1 time per week
- ☐ 2 times per week
- ☐ 3 or more times per week
- ☐ I don't know

Section E.2. Food and beverages for school age children.

☐ My site does not serve school age children. ⟶ If checked, go to Question #51.

46. What type of milk does your site serve to school age children? (Select all that apply.)

- ☐ Whole Milk
- ☐ Reduced-Fat (2%) Milk
- ☐ Low-Fat (1%) Milk
- ☐ Fat-Free (Skim) Milk
- ☐ I don't know

47. How often does your site serve flavored milk to school age children?

- ☐ Never
- ☐ Once a day
- ☐ Twice a day
- ☐ More than twice a day
- ☐ I don't know

48. How often does your site serve 100% juice as labeled on the package to school age children as part of a meal and/or snack?

- ☐ Never
- ☐ 1 time per week
- ☐ 2 times per week
- ☐ 3 or more times per week
- ☐ I don't know

49. How often does your site serve fruit drinks or fruit-flavored beverages other than 100% fruit juice such as Sunny Delight, Hi-C, Kool Aid, or lemonade to school age children?

- ☐ Never
- ☐ 1 time per week
- ☐ 2 times per week
- ☐ 3 or more times per week
- ☐ I don't know

50. How often does your site serve regular soda/pop (such as Sprite, orange soda, grape soda, cola) to school age children?

- ☐ Never
- ☐ 1 time per week
- ☐ 2 times per week
- ☐ 3 or more times per week
- ☐ I don't know

Section F. Revised child care regulations.

51. Are you familiar with the <u>revised</u> Colorado child care regulations for nutrition and physical activity that went into effect in February 2016?

- ☐ Yes → If yes, go to Question #52.
- ☐ No → If NO, go to Question #59
- ☐ I don't know → If I DON'T KNOW, go to Question #59.

52. To what extent do you feel that your site was <u>prepared to make changes</u> so that your site met the <u>revised</u> state regulations?

- ☐ Very Much
- ☐ Somewhat
- ☐ Not at all
- ☐ I don't know

53. When did your site <u>begin to implement</u> the <u>revised</u> state regulations related to nutrition and physical activity in your child care site?

- ☐ Prior to February 2016
- ☐ March 2016-December 2016
- ☐ January 2017 or later
- ☐ No changes needed. We were already compliant.
- ☐ I don't know

54. To what extent did the <u>revised</u> state regulations related to nutrition and physical activity require more work and resources related to <u>staffing</u>?

- ☐ Very Much
- ☐ Somewhat
- ☐ Not at all
- ☐ I don't know

55. To what extent did the <u>revised</u> state regulations related to nutrition require more work and resources related to <u>kitchen facilities</u>?

- ☐ Very Much
- ☐ Somewhat
- ☐ Not at all
- ☐ I don't know

56. Did the <u>revised</u> state regulations related to physical activity require your center to purchase additional <u>playground or activity equipment</u>? If YES, what type of equipment?

- ☐ Yes (please indicate) ______________________________
- ☐ No
- ☐ I don't know

57. To what extent do you feel that your site <u>needed help to implement</u> the <u>revised</u> state regulations related to nutrition and physical activity in your child care site?

- ☐ Very Much
- ☐ Somewhat
- ☐ Not at all
- ☐ I don't know

58. Did you get technical assistance from any agency or organization to implement the <u>revised</u> state regulations related to nutrition and physical activity? If YES, what agency or organization provided technical assistance?

- ☐ Yes (please indicate) ________________________________
- ☐ No
- ☐ I don't know

59. What is your name? (optional)

____

Thank you for your participation!

Please return this survey in the postage paid, enclosed envelope.

Upon receipt of the completed survey, we will send you a $20 gift card to Amazon.com.
